# Association of ITM2A rs1751094 polymorphism on X chromosome in Korean pediatric patients with autoimmune thyroid disease

**DOI:** 10.1002/iid3.800

**Published:** 2023-03-14

**Authors:** Won K. Cho, In‐Cheol Baek, Sung E. Kim, Mirae Kim, Tai‐Gyu Kim, Byung‐Kyu Suh

**Affiliations:** ^1^ Department of Pediatrics, College of Medicine, St. Vincent's Hospital The Catholic University of Korea Seoul Korea; ^2^ Catholic Hematopoietic Stem Cell Bank, College of Medicine The Catholic University of Korea Seoul Korea; ^3^ Department of Pediatrics, Incheon St. Mary's Hospital, College of Medicine The Catholic University of Korea Seoul Korea; ^4^ Department of Microbiology, College of Medicine The Catholic University of Korea Seoul Korea; ^5^ Department of Pediatrics, College of Medicine, Seoul St. Mary's Hospital The Catholic University of Korea Seoul Korea

**Keywords:** autoimmune thyroid disease, female‐predominant, intractable Grave's disease, ITM2A, susceptibility

## Abstract

**Background:**

Autoimmune thyroid disease (AITD) manifests with a female predominance, and much attention has been directed towards the integral membrane protein 2 A (ITM2A) gene located on the X chromosome.

**Methods:**

In a study of 166 pediatric patients with autoimmune thyroid disease (AITD), the ITM2A rs1751094 single‐nucleotide polymorphism (SNP) was genotyped. The sample comprised 143 females and 23 males, with 67 patients diagnosed with Hashimoto chronic thyroiditis (HD) and 99 with Graves' disease (GD). In the 99 GD patients, 49 (49.5%) exhibited thyroid‐associated ophthalmopathy (TAO). Among the 85 GD patients, 70.6% (60/85) were considered intractable GD. The results were compared to those from 198 healthy Korean individuals, including 97 females and 101 males.

**Results:**

The frequency of the rs1751094 C allele and CC/AC genotype were higher in AITD, GD and HD patients compared to controls, while the frequency of the A allele and AA genotype were lower. The results were more pronounced in female AITD and GD patients compared to male patients. The association was also found in intractable GD and TAO patients. Target SNP fits Hardy–Weinberg equilibrium.

**Conclusions:**

These findings indicate that the ITM2A gene polymorphism on the X chromosome may contribute to the immunological basis of female‐predominant AITD in Korean children.

## Graphical abstract

we found that ITM2A rs1751094 CC was disease‐susceptible and rs1751094 AA was disease‐protective genotypes in autoimmune thyroid disease (AITD) patient groups. These results suggest that polymorphism in ITM2A gene on the X chromosome play a role in the immunopathogenesis of female‐dominant AITD in Korean children.

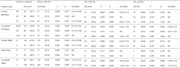

## 1 INTRODUCTION

Individuals with a genetic susceptibility may develop autoimmune thyroid disease (AITD) when exposed to environmental triggers, such as infection, iodine, or stress.^1^ Genetics play a significant role in the pathogenesis of AITD, and the genetic susceptibility might pose a greater concern in early‐onset cases compared to late‐onset cases of AITD.^2^ The Danish twin study has reported the role of heritability to Graves' disease (GD) development as 79%.^3^ AITD is composed of two main clinical forms, GD and Hashimoto chronic thyroiditis (HD), and is characterized by the infiltration of T and B cells into the thyroid and the production of antibodies directed against thyroid antigens.^4^ GD and HD both initiate with autoimmune responses targeted at the thyroid gland.^4^, ^5^ The occurrence of GD and HD within the same lineage suggests a shared genetic basis between the two conditions.^6^, ^7^, ^8^

Taiwan's nationwide cohort studies suggest a potential link between AITD, including both GD and HD, and increased risk of developing thyroid, breast, and colon cancers later in life,^9^, ^10^ although more research is needed to firmly establish this connection. Some patients with GD exhibit improvement through antithyroid drugs, while others experience relapse and turn to thyroidectomy or radioiodine ablation as alternative treatments.^11^ Among extra thyroidal manifestations, thyroid associated ophthalmopathy (TAO) is significantly associated with patient quality of life (QoL).^12^

The dominance of women in autoimmune diseases has been well established, and the most notable disparity in prevalence between the sexes is seen in AITD, with more than 80% of cases occurring in women.^13^, ^14^ The underlying mechanisms responsible for this sex difference are not well understood. However, it is suggested that genetic factors on the X chromosome could play a role in increasing disease susceptibility.^13^ The X chromosome possesses a substantial gene complement, estimated to be around 1000 genes, compared with the Y chromosome has a comparatively limited number of genes, estimated at around 100. These include genes implicated in the immune system. Some research suggests that direct genetic difference of many immune‐related genes in X chromosomes are associated with sex differences in immune response and disease prevalence.^15^ The presence of polymorphic genes on the X chromosome is of particular interest due to their varying allelic and genotype frequencies between females and males, which may result in distinct phenotypic effects.^16^ While women carry two copies of the X chromosome, the balancing of allelic dosages between females and males is achieved through the process of X chromosome inactivation (XCI), which silences one of the two X chromosomes randomly present in women.^17^ However, about 15%–20% of X chromosome‐linked genes escape XCI.^18^ In addition, these XCI escape genes having a lack of dosage compensation with Y chromosome are supposed to be related to the occurrence of autoimmune diseases.^13^ The current literature highlights the involvement of several XCI escape genes in the development of autoimmune disorders.^19^ The integral membrane protein 2A (ITM2A), a transcriptional target of GATA3, has also been reported as an XCI escapee in the majority of women.^20^

Recent studies have demonstrated correlations between genes located on the X chromosome and AITD. Our research has revealed that polymorphisms in the IL‐1 receptor‐associated kinase‐1 (IRAK1) and G protein‐coupled receptor 174 (GPR174) genes, located on the X chromosome, are associated with AITD in Korean children.^21^, ^22^, ^23^, ^24^ Some reports suggest that ITM2A is probably involved in the immunologic pathogenesis of GD.^25^ However, the biologic role of ITM2A in AITD remains largely unknown, and as far as we know, there is no prior information available regarding the correlation between ITM2A polymorphisms and AITD in Korean pediatric population. In this study, we examined the association of ITM2A rs1751094 polymorphism with AITD in Korean children.

## 2 MATERIALS AND METHODS

### 2.1 Subjects

The current investigation is a registry study that did not include any interventions. Patients who were diagnosed with either GD or HD at the pediatric endocrine clinics of Seoul St. Mary's and St. Vincent's Hospitals were included in this study. The exclusion criteria were patients who were diagnosed with other autoimmune diseases, hematologic diseases, or endocrine diseases or those who had an insufficient blood sample for genetic analysis. From March 2009 to August 2021, 234 individuals diagnosed with AITD consented to participate in the study. Of these 234 subjects, 68 were excluded due to having an insufficient blood sample for genetic analysis. Ultimately, our study included 166 pediatric patients (142 females and 24 males) diagnosed with AITD (67 HD and 99 GD cases). The mean age (±SD) of GD patients at enrollment was 14.4 ± 3.5 years, and HD patients were 13.8 ± 3.5 years. In GD patients, 49 patients (49/99, 49.5%) had thyroid‐associated ophthalmopathy (TAO). Among the 85 GD patients followed for at least 2 years, 60 patients (60/85, 70.6%) were intractable GD (Table 1). For the control group, a total of 198 healthy Korean adults without a history of autoimmune thyroid disease (AITD) were selected, consisting of 97 females and 101 males, and were genetically unrelated. We checked that there was clinically no other disease of the control through a medical examination by interview. The participants of the control group were primarily composed of students and employees from the Catholic University of Korea's Medical College and from a hematopoietic stem cell transplantation center. The students in the group were generally considered to be in good health without any significant health problems. All individuals in the study agreed to participate voluntarily after being fully informed, and the genetic study was cleared by the Institutional Review Board of the Catholic University of Korea (having IRB numbers KC09FISI0042 and MC13SISI0126).

**Table 1:** Characteristics of 166 autoimmune thyroid disease (AITD) patients and controls.

	AITD (*n* = 166)		Controls (*n* = 198)
	GD (*n* = 99)	HD (*n* = 67)	
Female, *n* (%)	80 (80.8%)	63 (94.0%)	97 (49.0%)
Age at enrollment (years)	14.4 ± 3.5	13.8 ± 3.5	
Age at diagnosis (years)	12.3 ± 3.1	11.1 ± 3.0	
Follow up periods (years)	7.8 ± 4.7	9.7 ± 4.4	
Goiter, *n* (%)	84 (84.8%)	38 (56.7%)	
T3 at diagnosis, 0.78–1.82 ng/mL	4.01 ± 2.31	1.31 ± 0.53	n.d.
Free T4 at diagnosis, 0.85–1.86 ng/dL	3.36 ± 1.45	1.13 ± 0.69	n.d.
TSH at diagnosis, 0.17–4.05 mIU/L	0.032 ± 0.044	29.30 ± 56.82	n.d.
TSHR Ab positive at diagnosis	97 (98.0%)		
Tg Ab positive at diagnosis		49 (73.1%)	
TPO Ab positive at diagnosis		56 (83.6%)	
Clinically evident TAO (NOSPECS class II or higher), *n* (%)	49 (49.5%)		
Intractable, *n* (%)	60/85 (70.6%)		
HD condition at diagnosis			
Euthyroid state		18 (26.9%)	
Subclinical hypothyroid state		21 (31.3%)	
Overt hypothyroid state		23 (34.3%)	
Hyperthyroid state		5 (7.5%)	

*Note*: Data are presented as mean ± SD or *n* (%). Remission, consistent with the improvement of clinical features and restoration of euthyroidism or induction of hypothyroidism after ATD therapy.

Abbreviations: AITD, autoimmune thyroid diseases; GD, Graves' disease; HD, Hashimoto's disease; n.d., not done; TSH, thyroid stimulating hormone; TSHR Ab, TSH receptor antibody; TPO, thyroid peroxidase; TAO, thyroid associated ophthalmopathy.

The diagnosis of HD was established when a minimum of three criteria from Fisher's list were satisfied, including: (1) the presence of a goiter, (2) a diffuse goiter with decreased uptake on a thyroid scan, (3) the detection of circulating thyroglobulin and/or microsomal autoantibodies, and (4) hormonal evidence of hypothyroidism.^26^ The diagnosis of GD was established through hyperthyroidism and supporting biochemistry, such as the presence of a goiter, high levels of ^131^I uptake in the thyroid, positive TSH receptor antibodies, and elevated levels of thyroid hormones.^11^ Remission in GD was defined as the resolution of symptoms and the return to a euthyroid state or the induction of hypothyroidism through ATD treatment. Intractable GD was defined as the persistence of hyperthyroidism despite 2 years of ATD therapy, a recurrence of symptoms after discontinuing ATD, or undergoing ATD treatment for at least 5 years.^27^, ^28^, ^29^ The diagnosis of TAO was established based on typical clinical symptoms and was classified using the classification system established by the American Thyroid Association Committee. Individuals who showed changes in soft tissue, proptosis, extraocular muscle dysfunction, or a mixture of these symptoms were deemed to have TAO.^30^ Subjects who displayed no symptoms or only a lid lag sign were placed in the non‐TAO group.^31^

### 2.2 DNA extraction

The genomic DNA was obtained from a 4 mL sample of peripheral blood that had been combined with ethylenediaminetetraacetic acid by utilizing TIANamp Genomic DNA Extraction Kits (manufactured by Tiangen Biotech Corporation) in accordance with the manufacturer's protocol. The DNA solution was adjusted to a concentration of 100 ng/μL, and was preserved at −20°C. The samples were then utilized as a template for polymerase chain reaction (PCR) genotyping.^32^

### 2.3 Target gene primer design and PCR

Genomic DNA was obtained from multiple samples, and the pediatric patient and control groups with AITD were suitable for analysis using a PCR template of 50 ng or lower. The primers for the ITM2A gene (rs1751094: A > C) are presented in Table 2. PCR amplifications were performed in 50 µL of reaction mixtures in 96‐well thin walled trays (Nippon Genetics). The reaction mixtures consisted of 10 μmol/L target‐specific primers, 150–300 ng genomic DNA, 1× buffer (60 mmol/L Tris‐Cl, 15 mmol/L ammonium sulfate, and 100 mmol/L MgCl2), 250 μmol/L dNTPs (dATP/dGTP/dCTP/dTTP, 250 μmol/L), and 1 U Taq DNA polymerase (Bioprince, Enzynomics).^33^ In PCR, the extracted genomic DNA was amplified in a ProFlex 96‐Well PCR System (Thermo Fisher Scientific) using the following PCR conditions: a preliminary step of 1 cycle at 95°C for 15 min followed by 40 cycles of denaturation at 94°C for 30 s, annealing at 63°C for 90 s, and extension at 72°C for 30 s. The final extension was performed at 60°C for 30 min.^22^

**Table 2:** Oligonucleotide sequences of primers for multiplex PCR amplifications.

Gene	SNP	Position (hg19)	Direction	Sequence (5′–3′)	Span (bp)*	Length	amplicon size
ITM2A	rs1751094	78,616,350	R	AAACAACGGATGGAATTTATTGTCAGGA	76	28	131
			F	TGGCATTGCTTGTTTTTTGAAACTGAA		27	

Abbreviations: PCR, polymerase chain reaction; SNP, single nucleotide polymorphism.

### 2.4 Sequencing

Sanger sequencing was performed using a Big Dye Terminator v3.1 (Amersham Pharmacia), and reactions were analyzed with ABI PRISM 3730XL analyzer (PE Applied Biosystems). Sequencing data was analyzed using the FinchTV 1.4 software (Geospiza, Inc.).

### 2.5 Statistical analysis

The allele frequencies were calculated using Microsoft Office Excel. The statistical significance was assessed using the *χ* ^2^ test and Fisher's exact test. The *p* values were adjusted by the Bonferroni method, and odds ratio (OR) was calculated using Haldane's modification of Woolf's method.^34^, ^35^ The statistical significance was considered at a level of <.05 after correction. The data from the case and control groups were analyzed by sex‐stratified groups or as a combined group. The Hardy–Weinberg equilibrium of each SNP in ITM2A on X chromosome was evaluated using the method proposed by Graelman^29^ and using the Haploview software version 4.2.^36^ The presence of linkage disequilibrium (LD) between polymorphisms was evaluated using Haploview 4.2.^36^

A sample size of 297 participants is required to attain 80% statistical power and 5% type 1 error, based on the 22% MAF of ITM2A SNPs, a dominant model, and a predicted prevalence of GD at 2.76 per 1000,^37^ using an unmatched case–control design. The power of our study was calculated based on an available sample size of 99 GD cases and 198 controls. The power for ITM2A rs1751094 were 0.8 when OR was 2.0. Sample size and power estimation were performed using Quanto 1.2.4 software (available at preventivemedicine.usc.edu in Los Angeles, CA).

## 3 RESULTS

### 3.1 Comparison of genotype and allele frequencies of ITM2A rs1751094 on the X chromosome in AITD patients and controls

Target SNP fits HWE (Table S1). In AITD patients (*n* = 166; *F* = 143, *M* = 23), the genotype and allele frequencies of rs1751094 AC [OR = 2.5 (95% confidence interval [CI], 1.6–3.8), corrected *p* (*p*c) = .000], C [OR = 2.0 (95%CI, 1.5–2.8), *p*c = .000] were higher and those of AA/A [OR = 0.3 (95%CI, 0.2–0.5), *p*c = .000], A [OR = 0.5 (95%CI, 0.4–0.7), *p*c = .000] were lower than those in controls (*n* = 198; *F* = 97, *M* = 101) (Table 3). When cases and controls were analyzed by sex, in female AITD patients (*F* = 143), those of rs1751094 CC [OR = 2.4 (95%CI, 1.2–4.8), *p*c = .031], C [OR = 1.8 (95%CI, 1.3–2.6), *p*c = 0.003] were higher and those of rs1751094 AA [OR = 0.5 (95%CI, 0.3–0.8), *p*c = 0.027], A [OR = 0.6 (95%CI, 0.4–0.8), *p*c = 0.003] were lower than female controls (*F* = 97) (Table 3).

**Table 3:** Genetic influence of ITM2A rs1751094 on X chromosome in AITD patients.

	Control *n* = 198 (%)			AITD *n* = 166 (%)					GD *n* = 99 (%)					HD *n* = 67 (%)				
Analysis type		F97 M101		F143 M23		*p*	*p*c	OR (95CI)	F80 M19		*p*	*p*c	OR (95CI)	F63 M4		*p*	*p*c	OR (95CI)
Female genotype	AA	36	(37.1)	31	(21.7)	.009	.027	0.5 (0.3–0.8)	13	(16.3)	.002	.006	0.3 (0.2–0.7)	18	(28.6)	.264	.793	NA
	AC	48	(49.5)	73	(51.0)	.812	2.436	NA	27	(33.8)	.035	.105	NA	33	(52.4)	.720	.161	NA
	CC	13	(13.4)	39	(27.3)	.010	.031	2.4 (1.2–4.8)	40	(50.0)	.000	.000	6.5 (3.1–13.4)	12	(19.0)	.337	1.010	NA
Combined Genotype	AA/A	107	(54.0)	43	(25.9)	.000	.000	0.3 (0.2–0.5)	23	(23.2)	.000	.000	0.3 (0.1–0.4)	20	(29.9)	.001	.002	0.4 (0.2–0.7)
	AC	48	(24.2)	73	(44.0)	.000	.000	2.5 (1.6–3.8)	27	(27.3)	.571	1.713	NA	33	(49.3)	.000	.000	3.0 (1.7–5.4)
	CC/C	43	(21.7)	50	(30.1)	.067	.201	NA	49	(49.5)	.000	.000	3.5 (2.1–5.9)	14	(20.9)	.887	2.662	NA
Female Allele	A	120	(61.9)	135	(47.2)	.002	.003	0.6 (0.4–0.8)	53	(33.1)	.000	.000	0.3 (0.2–0.5)	69	(54.8)	.207	.415	NA
	C	74	(38.1)	151	(52.8)	.002	.003	1.8 (1.3–2.6)	107	(66.9)	.000	.000	3.3 (2.1–5.1)	57	(45.2)	.207	.415	NA
Male Allele	A	71	(70.3)	12	(52.2)	.095	.191	NA	10	(52.6)	.131	.263	NA	2	(50.0)	.387	.774	NA
	C	30	(29.7)	11	(47.8)	.095	.191	NA	9	(47.4)	.131	.263	NA	2	(50.0)	.387	.774	NA
Combined Allele	A	191	(64.7)	147	(47.6)	.000	.000	0.5 (0.4–0.7)	63	(35.2)	.000	.000	0.3 (0.2–0.4)	71	(54.6)	.048	.096	0.7 (0.4–1.0)
	C	104	(35.3)	162	(52.4)	.000	.000	2.0 (1.5–2.8)	116	(64.8)	.000	.000	3.4 (2.3–5.0)	59	(45.4)	.048	.096	1.5 (1.0–2.3)

Abbreviations: AITD, autoimmune thyroid disease; CI, confidence interval; GD, Graves' disease; HD, Hashimoto chronic thyroiditis; OR, odds ratio.

### 3.2 Comparison of genotype and allele frequencies of ITM2A rs1751094 on the X chromosome in GD patients and controls

In GD patients (*n* = 99; *F* = 80, *M* = 19), the genotype and allele frequencies of rs1751094 CC/C [OR = 3.5 (95%CI, 2.1–5.9), *p*c = .000], C [OR = 3.4 (95%CI, 2.3–5.0), *p*c = .000] were higher and those of AA/A [OR = 0.3 (95%CI, 0.1–0.4), *p*c = .000], A [OR = 0.3 (95%CI, 0.2–0.4), *p*c = .000] were lower than controls (*n* = 198; *F* = 97, *M* = 101). When cases and controls were analyzed by sex, in female GD patients (*F* = 80), those of rs1751094 CC [OR = 6.5 (95%CI, 3.1–13.4), *p*c = .000], C [OR = 3.4 (95%CI, 2.3–5.0), *p*c = .000] were strongly higher and those of rs1751094 AA [OR = 0.3 (95%CI, 0.2–0.7), *p*c = .006], A [OR = 0.3 (95%CI, 0.2–0.4), *p*c = 0.000] were lower than female controls (*F* = 97) (Table 3).

In intractable GD patients (*n* = 60; F = 46, M = 14), those of rs1751094 AC [OR = 2.2 (95% CI, 1.2–4.1), corrected *p* (*p*c) = .026], C [OR = 2.5 (95%CI, 1.6–3.9), *p*c = .000] were higher and AA/A [OR = 0.2 (95%CI, 0.1–0.5), *p*c = .000], A [OR = 0.4 (95%CI, 0.3–0.6), *p*c = 0.000] were lower than controls (*n* = 198; *F* = 97, *M* = 101) (Tables 4 and 5). When cases and controls were analyzed by sex, in female intractable GD patients (*F* = 46), those of rs1751094 CC [OR = 2.8 (95% CI, 1.2–6.7), corrected *p* (*p*c) = .045], C [OR = 2.2 (95%CI, 1.3–3.7), *p*c = .004] were higher and AA [OR = 0.3 (95%CI, 0.1–0.8), *p*c = 0.023], A [OR = 0.5 (95%CI, 0.3–0.8), *p*c = .004] were lower than female controls (*F* = 97). In GD remission patients (*n* = 25; *F* = 21, *M* = 4), there was no significant difference in allele and genotype frequency of rs1751094 with controls at a *p*c level.

**Table 4:** Genotype (2n) influence of ITM2A rs1751094 on X chromosome in GD patients with TAO or intractable group.

Analysis	Control *n* = 198 (%)			GD intractable *n* = 60 (%)					GD remission *n* = 25 (%)					TAO *n* = 49 (%)					GD non‐TAO *n* = 50 (%)				
		F97 M101		F46 M14		*p*	*p*c	OR (95CI)	F21 M4		*p*	*p*c	OR (95CI)	F37 M12		*p*	*p*c	OR (95CI)	F43 M7		*p*	*p*c	OR (95CI)
Female geno type	AA	36	(37.1)	7	(15.2)	.008	.023	0.3 (0.1–0.8)	5	(23.8)	.246	.737	NA	4	(10.8)	.003	.009	0.2 (0.1–0.6)	9	(20.9)	.059	.176	NA
	AC	48	(49.5)	25	(54.3)	.587	1.760	NA	9	(42.9)	.582	1.745	NA	16	(43.2)	.518	1.554	NA	23	(53.5)	.662	1.986	NA
	CC	13	(13.4)	14	(30.4)	.015	.045	2.8 (1.2–6.7)	7	(33.3)	.027	.082	NA	17	(45.9)	.000	.000	5.5 (2.3–13.1)	11	(25.6)	.078	.233	NA
CombiNed	AA/A	107	(54.0)	13	(21.7)	.000	.000	0.2 (0.1–0.5)	9	(36.0)	.089	.267	NA	11	(22.4)	.000	.000	0.2 (0.1–0.5)	12	(24.0)	.000	.000	0.3 (0.1–0.5)
	AC	48	(24.2)	25	(41.7)	.009	.026	2.2 (1.2–4.1)	9	(36.0)	.204	.612	NA	16	(32.7)	.229	.687	NA	23	(46.0)	.002	.007	2.7 (1.4–5.1)
	CC/C	43	(21.7)	22	(36.7)	.019	.058	NA	7	(28.0)	.478	1.434	NA	22	(44.9)	.001	.003	2.9 (1.5–2.7)	15	(30.0)	.216	.649	NA

Abbreviations: CI, confidence interval; GD, Graves' disease; TAO, thyroid‐associated ophthalmopathy; OR, odds ratio.

**Table 5:** Allele(n) influence of ITM2A rs1751094 on X chromosome in GD patients with TAO or intractable group.

	Control *n* = 198 (%)			GD intractable *n* = 60 (%)					GD remission *n* = 25 (%)					TAO *n* = 49 (%)					GD non‐TAO *n* = 50 (%)				
		F97 M101		F46 M14		*p*	*p*c	OR (95CI)	F21 M4		*p*	*p*c	OR (95CI)	F37 M12		*p*	*p*c	OR(95CI)	F43 M7		*p*	*p*c	OR (95CI)
Female allele	A	120	(61.9)	39	(42.4)	.002	.004	0.5 (0.3–0.8)	19	(45.2)	.047	.094	NA	24	(32.4)	.000	.000	0.3 (0.2–0.5)	41	(47.7)	.027	.054	NA
	C	74	(38.1)	53	(57.6)	.002	.004	2.2 (1.3–3.7)	23	(54.8)	.047	.094	NA	50	(67.6)	.000	.000	3.4 (1.9–6.0)	45	(52.3)	.027	.054	NA
Male allele	A	71	(70.3)	6	(42.9)	.041	.082	NA	4	(100.0)	.197	.394	NA	7	(58.3)	.397	.794	NA	3	(42.9)	.131	.261	NA
	C	30	(29.7)	8	(57.1)	.041	.082	NA	0	(0.0)	.197	.394	NA	5	(41.7)	.397	.794	NA	4	(57.1)	.131	.261	NA
Combin allele	A	191	(64.7)	45	(42.5)	.000	.000	0.4 (0.3–0.6)	23	(50.0)	.054	.109	NA	31	(36.0)	.000	.000	0.3 (0.2–0.5)	44	(47.3)	.003	.005	0.5 (0.3–0.8)
	C	104	(35.3)	61	(57.5)	.000	.000	2.5 (1.6–3.9)	23	(50.0)	.054	.109	NA	55	(64.0)	.000	.000	3.3 (2.0–5.4)	49	(52.7)	.003	.005	2.0 (1.3–3.3)

Abbreviations: CI, confidence interval; GD, Graves' disease; TAO, thyroid‐associated ophthalmopathy; OR, odds ratio.

In TAO patients (*n* = 49; *F* = 37, *M* = 12), those of rs1751094 CC/C [OR = 2.9 (95% CI, 1.5–2.7), corrected *p* (*p*c) = .003], C [OR = 3.3 (95%CI, 2.0–5.4), *p*c = .000] were higher and AA/A [OR = 0.2 (95%CI, 0.1–0.5), *p*c = .000], A [OR = 0.3 (95%CI, 0.2–0.5), *p*c = .000] were lower than controls (*n* = 198; *F* = 97, *M* = 101). When cases and controls were analyzed by sex, in female TAO patients (*F* = 37), those of rs1751094 CC [OR = 5.5 (95% CI, 2.3–13.1), corrected *p* (*p*c) = .000], C [OR = 3.4 (95%CI, 1.9–6.0), *p*c = .000] were higher and AA [OR = 0.2 (95%CI, 0.1–0.6), *p*c = 0.009], A [OR = 0.3 (95%CI, 0.2–0.5), *p*c = .000] were lower than controls (*F* = 97). In non‐TAO patients (*n* = 50; *F* = 43, *M* = 7), those of rs1751094 AC [OR = 2.7 (95% CI, 1.4–5.1), corrected *p* (*p*c) = .007], C [OR = 2.0 (95%CI, 1.3–3.3), *p*c = 0.000] were higher and AA/A [OR = 0.3 (95%CI, 0.1–0.5), *p*c = .000], A [OR = 0.5 (95%CI, 0.3–0.8), *p*c = 0.005] were lower than controls (*n* = 198; *F* = 97, *M* = 101).

### 3.3 Comparison of genotype frequencies of ITM2A rs1751094 on the X chromosome in HD patients and controls

In HD patients (*n* = 67; *F* = 63, *M* = 4), the genotype frequency of rs1751094 AC [OR = 3.0 (95% CI, 1.7–5.4), *p*c = 0.000] was higher and that of AA/A [OR = 0.4 (95%CI, 0.2–0.7), *p*c = 0.002] was lower than in controls (*n* = 198; *F* = 97, *M* = 101) (Table 3).

## 4 DISCUSSION

In this study, ITM2A rs1751094 Polymorphism was discovered to be associated with AITD, GD and HD patients. The frequency of the rs1751094 C allele and CC/AC genotype were higher in AITD, GD and HD patients compared to controls, while the frequency of the A allele and AA genotype were lower. The results were more pronounced in female AITD and GD patients compared to male patients. The association was also found in intractable GD and TAO patients.

**Figure 1:**
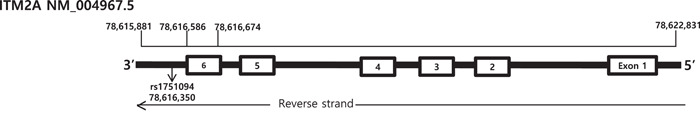
Genomic regions of Integral membrane protein 2A (ITM2A) NM_004967.5 from GRCh37.p13 (hg19). ITM2A rs1751094 is located at 3 prime untranslated region (3′UTR) variant, T > A,C,G. (https://www.ncbi.nlm.nih.gov/genome/gdv/browser/gene/?id=9452).

ITM2A is located at Xq21.1 (Figure 1) and predominantly expressed in CD4 T cells, and is induced during major histocompatibility complex‐mediated positive selection of double positive thymocytes.^38^ ITM2A, a 263‐amino acid type II transmembrane protein, is a member of the integral membrane protein family, which includes ITM2B and ITM2C and is part of the BRICHOS superfamily.^39^ ITM2A has been identified as playing a role in autoimmune disease and myogenic differentiation.^25^, ^40^ An in vivo study demonstrated that mice lacking Itm2a showed a reduced T helper cell‐mediated immune response.^38^ The immune response observed in GD is predominated by CD4 Th2 and exhibits an elevated CD4 + /CD8 + T cell ratio.^41^ Some reports suggest that ITM2A is probably implicated in the immunopathogenesis of GD.^25^ In addition, immune cells with rs1751094 CC expressed higher serum ITM2A levels than those with the rs1751094 AA genotypes (https://dice-database.org/eqtls/rs1751094#TREG_MEM_ITM2A). These results suggest that rs1751094 CC could be the disease susceptible genotype in AITD children.

ITM2A has been shown to be one of XCI escape genes.^20^ A restriction fragment‐length polymorphism (RFLP) study identified that mRNA transcripts of the ITM2A rs1751094 heterozygote genotype AC exhibited two distinct bands after digestion, suggesting that ITM2A rs1751094 can escape XCI in the PBMCs.^23^ The other study showed that ITM2A is biallelically expressed in plasmablasts, a B cell subset and is considered an XCI escapee.^42^ In females, at single cell levels, gene expression of heterozygous SNPs on XCI escape gene means that there are three distinct cell populations expressing exclusively one, the other and both alleles.^43^, ^44^ Consequently, female individuals are composed of cellular mosaics in their polymorphic XCI escape genes. These disparities in X‐linked gene expression between males and females may influence the phenotypic expression of X‐linked SNPs in various physiological and pathological immune responses.^16^

Clinically, some patients with GD achieve remission, but intractable GD patients consider total thyroidectomy or radioactive iodine (RAI) ablation.^11^ In spite of both total thyroidectomy and RAI being definite treatments with excellent therapeutic effects, side effects such as bleeding, vocal‐cord paralysis, tracheostomy, and hypocalcemia should be considered. In addition, RAI should be considered such as worsening of TAO and radiation exposure.^45^ Therefore, prediction of intractable GD at the beginning of treatment is necessary for tailored personalized treatment. Some reports suggested the predictive role of the absence of goiter, serum TSHR Ab level at time of GD diagnosis or shorter TSHR Ab normalization time.^11^, ^27^, ^46^ However, it is difficult to predict early onset intractable GD at the beginning of treatment. Genetically, intractable GD has been reported to correlate with TSHR gene polymorphisms.^47^ In this study, polymorphisms of ITM2A rs1751094 on the X chromosome were also associated with intractable GD, that is, with poor prognosis.

TAO, a prevalent autoimmune and inflammatory condition of the orbit, exhibits close association with GD.^48^ Recently, we reported that polymorphisms of MICA, TLR10, TLR4, TLR‐9, and IL‐12 genes are associated with non‐TAO GD group in Korean children.^49^, ^50^, ^51^, ^52^ In this study, polymorphisms in ITM2A rs1751094 on the X chromosome showed an association in the TAO group, and it was confirmed that it was also associated with extra thyroid manifestation.

As far as we know, this is the first report of an association between ITM2A rs1751094 and patients with AITD (https://www.ncbi.nlm.nih.gov/snp/rs1751094). The major strength of this study is the finding the association between ITM2A rs1751094 SNP and AITD, GD, intractable GD, GD‐TAO and HD in Korean pediatric patients. The strongest association was found in female GD patients. However, the limitations of this study include the small sample size and the fact that the control group was not evaluated through laboratory methods to rule out subclinical cases of AITD. In conclusion, these results suggest that the ITM2A gene polymorphism on the X chromosome may play a role in the immunological basis of female‐predominant AITD in Korean children.

## AUTHOR CONTRIBUTIONS

**Won Kyoung Cho**: Conceptualization (lead); writing—original draft (lead); formal analysis (lead); writing—review and editing (equal); methodology (supporting). **In‐Cheol Baek**: Methodology (lead); software (lead); writing—review and editing (equal). **Sung Eun Kim**: Conceptualization (supporting); writing—review and editing (equal). **Mirae Kim**: Methodology (supporting); software (supporting). **Tai‐Gyu Kim**: Conceptualization (supporting); writing—review and editing (equal). **Byung‐Kyu Suh**: Conceptualization (supporting); writing—review and editing (equal).

## CONFLICT OF INTEREST STATEMENT

The authors declare that they have no competing interests.

## ETHICS APPROVAL AND CONSENT TO PARTICIPATE

The study was approved by the Institutional Review Board of the Catholic University of Korea (IRB Number: KC09FISI0042, MC13SISI0126), and all participants gave informed consent for genetic study participation.

## Supporting information

Supplementary information.

## Data Availability

The datasets generated during and/or analyzed during the current study are not publicly available but are available from the corresponding author on reasonable request.
