# Supplementary material for: Association of ITM2A rs1751094 polymorphism on X chromosome in Korean pediatric patients with autoimmune thyroid disease
Source: Immun Inflamm Dis. 2023 Mar 14;11(3):e800. doi: 10.1002/iid3.800 (PMC10013136; doi:10.1002/iid3.800)
Supplement: Supplementary file 1 — Supplementary information. [file IID3-11-e800-s001.docx]

Supplementary Table 1. Hardy Weinberg equilibrium for ITM2A rs1751094

1. With or without males

|  | Genotype and allele | | | | | HWp-value | |
| --- | --- | --- | --- | --- | --- | --- | --- |
|  |  |  |  |  |  |  |  |
|  | mA | mC | fAA | fAC | fCC | ALL | Female |
| Control | 71 | 30 | 36 | 48 | 13 | 0.319 | 0.63 |
| ITM2A (rs1751094 : A>C) | 12 | 11 | 31 | 73 | 39 | 0.863 | 0.77 |

Abbreviations: HW, Hardy Weinberg equilibrium; m, male; f, female.

1. Hardy Weinberg equilibrium for ITM2A rs1751094 with case/control analysis using Haploview

| Name | Position | ObsHET | PredHET | HWpval | %Geno | FamTrio | MendErr | MAF | Alleles |
| --- | --- | --- | --- | --- | --- | --- | --- | --- | --- |
| rs1751094 | 78,616,350 | 0.504 | 0.498 | 0.9775 | 100.0 | 0 | 0 | 0.469 | A:C |

Abbreviations: Position, the marker position specified; ObsHET, the marker's observed heterozygosity; PredHET, the marker's predicted heterozygosity; HWpval, the Hardy-Weinberg equilibrium p value; %Geno, the percentage of non-missing genotypes for this marker; FamTrio, the number of fully genotyped family trios for this marker; MendErr, the number of observed Mendelian inheritance errors; MAF, the minor allele frequency. The Chi square test for Hardy-Weinberg (HWE), using two degrees of freedom, was calculated in both sexes according to the following alleles and genotypes: in males, p and q are the number of males carrying respectively allele, while in female, p², 2pq and q² are the number of females carrying one of the probable respectively genotype. Two times analysis conducted by directly statistical analysis and Haploview software, version 4.2. [27, 31].
